# Initial Treatment Modalities in Patients with Newly Diagnosed Primary Lung Cancer in Japan

**DOI:** 10.3390/curroncol32010032

**Published:** 2025-01-07

**Authors:** Qingqing Hu, Kuan-Chih Huang, Ko Nakajo, Yongjing Zhang, Hong Qiu

**Affiliations:** 1 Global Epidemiology, Office of the Chief Medical Officer, Janssen Research & Development, LLC, Shanghai 200231, China; qhu9@its.jnj.com (Q.H.); yzhan247@its.jnj.com (Y.Z.); 2 Global Epidemiology, Office of the Chief Medical Officer, Janssen Research & Development, LLC, Taipei 104, Taiwan; thuang21@its.jnj.com; 3 Global Epidemiology, Office of the Chief Medical Officer, Janssen Research & Development, LLC, Tokyo 101-0065, Japan; knakajo@its.jnj.com; 4 Global Epidemiology, Office of the Chief Medical Officer, Janssen Research & Development, LLC, Titusville, NJ 08560, USA

**Keywords:** lung cancer, targeted therapy, checkpoint inhibitor, chemotherapy, Japan

## Abstract

Treatment for lung cancer continues to rapidly evolve. Here, we describe trends in the initial treatment of adults with newly diagnosed primary non-small-cell lung cancer in Japan. This retrospective cohort study used data from JMDC Inc. Claims Database from 2015 to 2023. Adults with lung cancer, confirmed using a combination of diagnosis, treatment, or procedure codes, were enrolled. A total of 9373 patients were included, with a mean age of approximately 59 years. The median time from diagnosis to treatment initiation ranged from 38 days in patients treated surgically to 25 days in patients with distant metastases. The observed trends were a decrease in the percentage of newly diagnosed patients with distant metastases, a decline in chemotherapy use in patients with early-stage disease, and in advanced disease, a more than doubling in the use of targeted therapy, including checkpoint inhibitors, while radiotherapy and chemotherapy tended to decrease. The observed changes in treatment were driven mainly by the increased use of targeted therapies including checkpoint inhibitors and are aligned with current treatment guidelines in Japan. The observation of fewer patients with distant metastases over time possibly indicates earlier detection. Additional research is needed to understand if new therapies are being extended to older and frail patients with lung cancer in Japan.

## 1. Introduction

Lung cancer is the leading cause of cancer death in Japan and was responsible for more than 83,000 deaths in 2022 [1]. Non-small-cell lung cancer (NSCLC) is the most common histologic type and accounts for 85% of all lung cancers. The management of lung cancer has evolved rapidly over the last decade. Improved detection rates, evidence-based changes in staging, improvements in radiation therapy, improved drug delivery systems, and the emergence of immunotherapies such as immune checkpoint inhibitors and other targeted treatments such as tyrosine kinase inhibitors (TKIs) have all contributed to better clinical outcomes for patients [2,3]. Japan is one of few countries that provides universal health insurance, and approved drugs are subsidized up to 70–90% depending on age [4]. As such, patients have ready access to a wide range of treatment options.

The Japan Lung Cancer Society guidelines recommend that all patients diagnosed with NSCLC should be tested for actionable biomarkers, including EGFR mutations, anaplastic lymphoma kinase rearrangements/fusion, and programmed cell death (ligand) 1 (PD-[L]1) expression [5,6]. In patients with stage III unresectable NSCLC and positive driver gene alterations, targeted agents are recommended for first-line treatment [7,8]. In patients without driver gene alterations, a combination of immune checkpoint inhibitors and platinum-based chemotherapy is recommended, regardless of PD-(L)1 status [7,8].

The first anti-PD-(L)1 antibody (nivolumab) was approved for advanced NSCLC in Japan in 2015, and the first TKI (gefitinib) was approved in 2002 [9,10]. Subsequently, second- and third-generation TKIs as well as numerous checkpoint inhibitors have been approved and are reimbursed for use in NSCLC in Japan [8].

While they do provide increased flexibility for patients, the growing number of medical therapeutic options increases the complexity of treatment decision-making. A database study conducted in Kyoto showed that between 2013 and 2019, treatment patterns for lung cancer changed, characterized by an increase in surgical procedures commensurate with earlier detection, an increase in medical costs, particularly for drug therapy, and improved survival [11]. In view of the rapid evolution of lung cancer management, we aimed to describe temporal trends in treatment practices and provide an up-to-date picture of first-line treatment patterns for NSCLC in real-world practice in Japan. These data could have utility for future policy development as new therapies for lung cancer continue to enter the market.

## 2. Materials and Methods

### 2.1. Study Design

This study was a retrospective cohort study using the Japan Medical Data Center (JMDC) database [12]. The study objective was to describe initial treatment episodes and their temporal trends in adults with NSCLC in Japan. Adult patients with confirmed primary malignant lung cancer between 1 January 2015 and 31 March 2023 and who received treatment for lung cancer were enrolled. The index date was the date of the first identified lung cancer diagnosis (International Classification of Disease 10th revision clinical modification [ICD-10-CM] codes C34.0-C34.9) in the database. Patients were to have at least six months of enrollment in the database before the index date, which was defined as the baseline period. The initial treatment episode was defined as the period in which the first single or multiple treatments were prescribed before a >90-day gap, until study’s end.

### 2.2. Data Source

The JMDC is an employment insurance claims database operating since 2005 that includes the medical records of non-government employees (approximately 80% of members) and their familial dependents up to the age of 75 years (approximately 20% of members). The database holds records for around 17 million persons, representing between 1 and 2% of the population of Japan. Data from claims relating to all inpatient, outpatient, and pharmacy healthcare services are captured. Available data include demographic information, diagnostic disease codes in ICD-10 format, records of hospitalization, and prescriptions. Personal identifiable information is encrypted to protect patient privacy.

The anonymized data were obtained from the database under license by Janssen Research & Development from JMDC Inc. (formerly Japan Medical Data Center Co., Ltd., Tokyo, Japan). As the data were anonymized by the data provider and do not include interventions or patient interactions, the study was exempted from obtaining written informed consent based on the Ethical Guidelines on Biomedical Research Involving Human Subjects (Ministry of Health, Labor and Welfare of Japan).

### 2.3. Study Population

The study population included all adults aged 18 years and older who had at least two diagnosis codes for primary lung cancer, and at least one procedure code and/or pharmacy code for a lung cancer treatment in the JMDC during the study period (Tables S1–S3). Only those patients with at least 6 months of enrollment in the database prior to the index date, and with 3 months of enrollment after the index date, were included. We excluded patients who had an ICD-10-CM C34.0-C34.9 code but who had a Japanese standard disease code of small-cell lung cancer, and patients with other types of cancer in the lung (carcinoid, adenoid cystic cancer, sarcomas, blastoma, and melanoma) [13]. Patients with secondary lung cancers were excluded by removing all individuals with at least two diagnosis codes for another primary cancer (except non-melanoma skin cancer) (ICD-10-CM: C00-C96, except C44) during the baseline period.

In the absence of TMN staging data in the claims-based database, we assessed disease status according to whether patients had received surgical treatment (Table S2) and/or whether they had metastases at diagnosis (Table S4) [14]. Patients were stratified into four mutually exclusive disease groups based on treatment guidelines [5]: the S (surgery) group which included patients with at least one record of lung surgery, capturing patients with early-stage disease in whom first-line treatment is usually surgery; the NS-NM (no surgery–no metastases) group which included patients with no record of surgery and no record of metastasis; the NS-RM (no surgery–regional metastases) group which included patients with no record of surgery but at least one record of regional metastasis (the NS-NM and NS-RM groups captured patients with unresectable locally advanced disease); and the NS-DM (no surgery–distant metastases) group which included patients with no record of surgery but at least one record of distant metastasis, capturing patients with unresectable, late-stage disease.

### 2.4. Statistical Analysis

The demographic characteristics of patients were described. Categorical variables were summarized using frequencies and percentages, and continuous variables with medians and first and third quartiles (Q1, Q3). Treatments were grouped into surgery, radiotherapy, chemotherapy, checkpoint inhibitors, or other targeted therapies. The time from diagnosis to treatment initiation, treatment duration, and the use of monotherapy or combination therapy during the first treatment episode were described. Raw data were used to visualize temporal trends in each group across the 2015–2016, 2017–2018, 2019–2020, and 2021–2022 calendar years.

Comorbidities present during the baseline period were assessed using Charlson co-morbidity index (CCI) scores and were obtained using ICD-10 codes [15].

## 3. Results

### 3.1. Patient Characteristics

**Figure 1:**
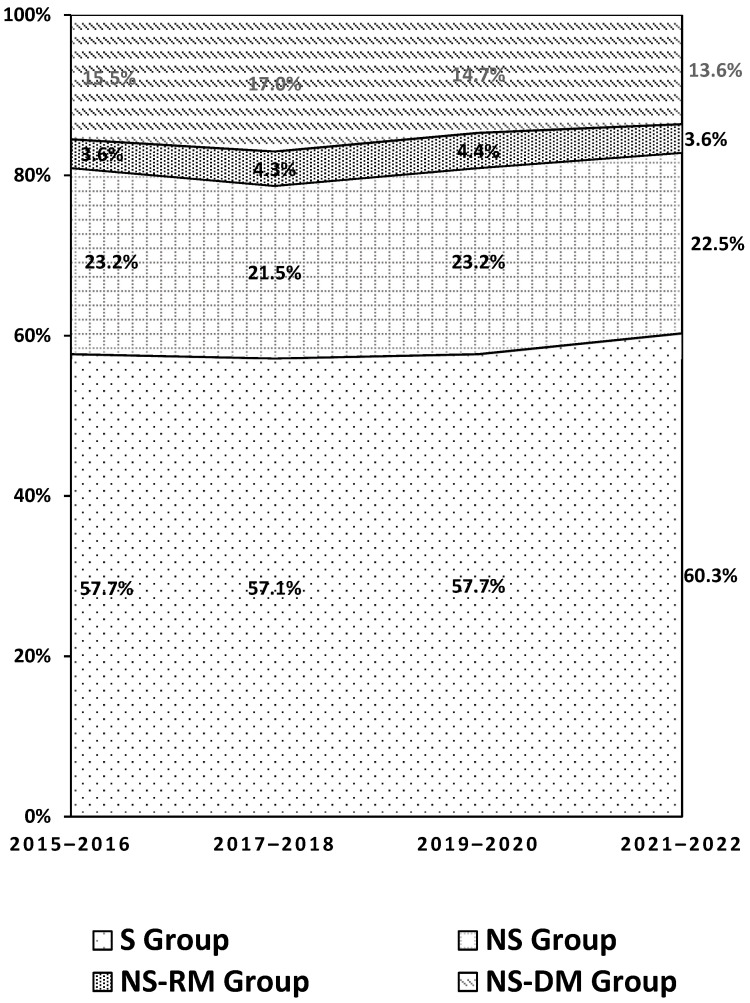
Distribution of patients with NSCLC by disease group, 2015–2022. S group: patients with at least one record of surgery; NS-NM group: patients with no record of surgery and no metastasis; NS-RM group: patients with no record of surgery but at least one record of regional metastasis; NS-DM group: patients with no record of surgery but at least one record of distant metastasis.

After the application of eligibility criteria, 9373 patients were included in the study (Figure S1). Of these, 5479 (58.5%) were in the S group, 2084 (22.2%) were in the NS-NM group, 375 (4.0%) were in the NS-RM group, and 1435 (15.3%) were in the NS-DM group. The percentage of patients with distant metastases at diagnosis decreased from 15.5% to 13.6% over the study period, but there was little change in the proportion of patients in other groups over the study period (Figure 1). Groups were similar in terms of demographic characteristics. Between 64.1% and 70.5% of patients in each group were male, and the median age was 59.59 to 60.94 years (Table 1). In each group, 61.5% to 67.5% of patients had a CCI score of 0, and 24.7% to 34.4% were smokers during the baseline period.

### 3.2. Time to Treatment Initiation and Treatment Duration

The median time from diagnosis to the initiation of the first lung cancer treatment was 38 days (Q1, Q3; 22, 58) in the S group, 34 days (19, 69.5) in the NS-NM group, 33 days (19, 56) in the NS-RM group, and 25 days (14, 40) in the NS-DM group (Table 2). Most patients (71.9% to 86.5% across all groups) started treatment within 60 days after diagnosis, and 58.1% of patients in the NS-DM group started within 30 days of diagnosis. The median duration of the initial treatment episode was 1 day in the S group, versus 196 days in the NS-NM, 238 days in the NS-RM, and 260 days in the NS-DM groups.

### 3.3. Initial Treatment Episode

**Figure 2:**
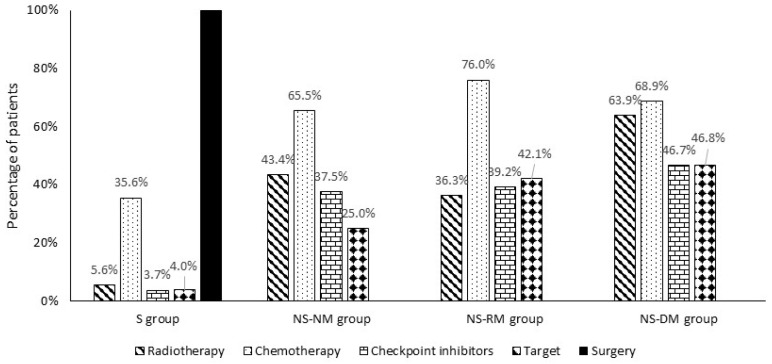
Therapies used during the initial treatment episode in patients with NSCLC during the study period, 2015–2022. S group: patients with at least one record of surgery; NS-NM group: patients with no record of surgery and no metastasis; NS-RM group: patients with no record of surgery but at least one record of regional metastasis; NS-DM group: patients with no record of surgery but at least one record of distant metastasis.

All patients in the S group received first-line treatment with surgery, and 59.3% received surgery alone, suggesting that a majority of patients in this group had early-stage disease (Figure 2, Table 3). Chemotherapy was administered to 35.6% of patients in the S group following surgery, for 27.5% of whom chemotherapy was the only systemic treatment. In the S group, 5.6% or less of patients also received radiotherapy, a checkpoint inhibitor, or other targeted therapy.

Chemotherapy was used by 65.5% and 76.0% of patients in the NS-NM and NS-RM groups, respectively, of which most was co-administered with a checkpoint inhibitor/targeted therapy (17.3% and 31.5% in the respective groups) or radiotherapy (31.5% and 32.8%). In patients treated with chemotherapy, 85.9% in the NS-NM group and 78.9% in the NS-RM group received concurrent chemotherapy and radiotherapy. Overall, radiotherapy was administered to 43.4% of patients in the NS-NM group and 36.3% in the NS-RM group, checkpoint inhibitors to 37.5% and 39.2%, and other targeted therapies to 25.0% and 42.1%, respectively (Figure 2, Table 3).

The use of radiotherapy was highest in the group with distant metastases (NS-DM, 63.9%); 17.4% of patients received radiotherapy alone, and 47.1% also received chemotherapy. In total, 46.7% of patients in this group received checkpoint inhibitors and 46.8% received other targeted therapy, usually combined with chemotherapy or radiotherapy.

Other systemic therapy (including immunosuppressants, hormones and related agents, or immunostimulants) was administered to 21.1% of patients in the NS-NM group, versus 8.0% in the NS-RM group and 4.6% in the NS-DM group (Table 3).

Concurrent chemoradiotherapy was identified when the first date of chemotherapy and the first date of RT occurred within a 0–30-day period or if the treatment period overlapped [16]. Sequential CRT was identified when there was a >30-day gap between the first date of chemotherapy and the first date of RT, or a period of overlap between chemotherapy and RT.

### 3.4. Temporal Trends in Initial Treatment Episodes

**Figure 3:**
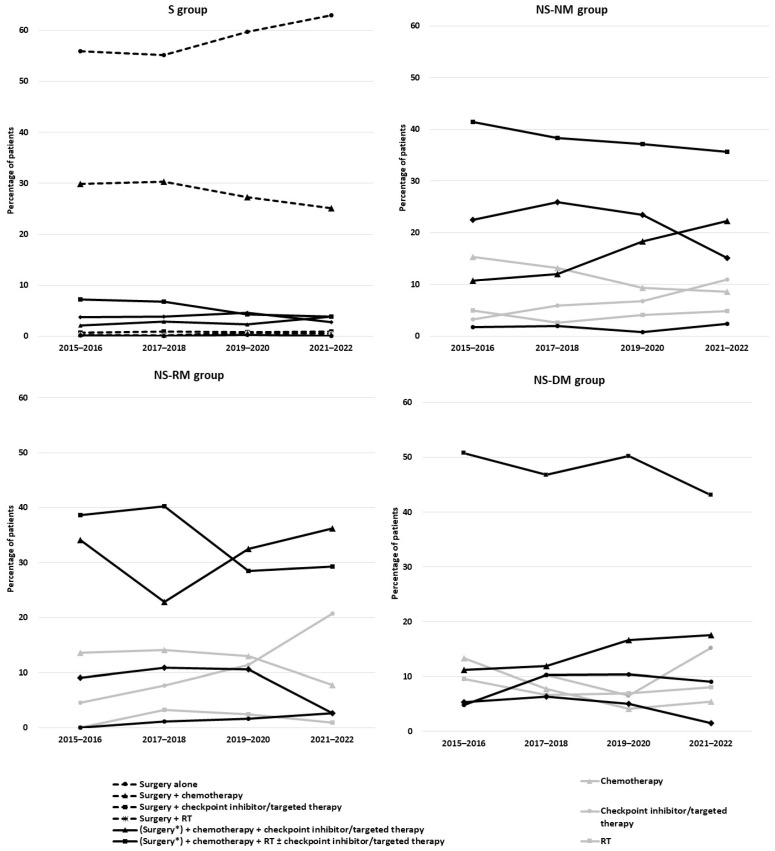
Trends in therapies used during initial treatment episodes for NSCLC, 2015–2022. PD-(L)1—programmed cell death (ligand) 1; RT—radiotherapy. S group: patients with at least one record of surgery; NS-NM group: patients with no record of surgery and no metastasis; NS-RM group: patients with no record of surgery but at least one record of regional metastasis; NS-DM group: patients with no record of surgery but at least one record of distant metastasis. * only patients in the S group.

In the S group, the percentage of patients who received surgery alone increased from 55.9% in 2015–2016 to 62.9% in 2021–2022, and the percentage who received chemotherapy along with surgery declined from 29.2% in 2015–2016 to 24.8% in 2021–2022 (Figure 3). There was little change in the use of checkpoint inhibitors, other targeted therapies, or radiotherapy over time.

By contrast, several marked changes in treatment patterns were observed in the other groups (Figure 3). Chemoradiotherapy with/without checkpoint inhibitors was the most frequently administered first-line treatment in the NS-NM, NS-RM, and NS-DM groups, but its use declined in all groups over the study period, from 41.4% to 35.7% in the NS-NM group, 38.6% to 29.3% in the NS-RM group, and 50.8% to 43.1% in the NS-DM group. The use of checkpoint inhibitors more than doubled from 16.1% in 2015–2016 to 47.6% in 2020–2022 in the NS-NM group, from 20.5% to 50.0% in the NS-RM group, and from 26.2% to 53.5% in the NS-DM group. The use of other targeted therapies increased in the NS-NM group from 22.5% in 2015–2016 to 29.5% in 2020–2022 and from 38.0% to 50.1% in the NS-DM group.

## 4. Discussion

We used the JMDC claims database to investigate real-world trends in the first-line treatment of NSCLC in Japan, where universal health insurance provides ready access to new therapies as they enter the market. The proportion of patients with evidence of distant metastases at diagnosis decreased by approximately 15% over the study period, suggesting earlier detection, although the proportion of patients with early-stage disease, represented in our study by the S group, showed little change over time. Most patients were treated within 60 days after their diagnosis, with a more rapid onset of treatment in the group with distant metastases at diagnosis. Our estimates are in line with the median time to treatment of 34 days in patients with lung cancer reported in a study using data from the national database of hospital-based cancer registries in Japan [17]. In groups that did not receive surgical treatment, the duration of the initial treatment episode was more than 6 months.

There was no evidence that the overall approach to the management of early-stage NSCLC changed dramatically during the study years, although the percentage of patients receiving chemotherapy decreased by approximately 16% over time, which could indicate earlier diagnosis with more patients undergoing curative surgical resection. By contrast, changes in the treatment of patients with more advanced disease were marked, mainly driven by the rapidly increasing availability of checkpoint inhibitors, other targeted therapies, and genetic testing, in line with Japanese guidelines [5,7]. The prevalence of EGFR mutations in Japanese patients with NSCLC is estimated to be 36.6% [18]. The rates of PD-L1 expression are less well described, but in one study, 26.6% of patients had a PD-L1 score of 50% or higher [19].

Our results differ from those reported by Shimamoto et al. in Kyoto between 2013 and 2018 [11], who showed an increase in surgical treatment over time, accompanied by a decreasing use of chemotherapy, with low rates of immune checkpoint inhibitor and targeted therapy use overall (around 5% of patients). The mean age of patients in the Kyoto study was 73 years, and almost 50% were aged 75 years and older, whereas 80% of members in the JMDC database are employees aged less than 65 years, and insurance coverage stops after the age of 75 years for family members. This is reflected by the mean age of our cohort being <60 years in each group and is likely to contribute to the differences observed.

Cancer staging data are not available from claims-based databases, so we used the initial treatment to define four stages as proxies for disease severity based on consensus guidelines [20]. The groups did not differ in terms of age and CCI score at baseline. We did not have access to other indicators of overall health and functioning such as Eastern Cooperative Oncology Group scores, which are expected to be lower in patients with advanced disease, and which could have partially validated our group definitions.

Nationwide data for the US from 2010 to 2016, which is before the start of our study, showed that in <65-year-olds, fewer patients with resectable disease received surgery or chemotherapy over time, while an increased percentage received radiotherapy [21]. The percentage of patients with stage IIIB disease receiving radiotherapy increased, while there was little change in the treatment of patients with stage IV disease.

The strengths of our study included the use of a large, longitudinal claims database that allowed the identification of a large cohort of Japanese patients diagnosed with lung cancer. The 8-year study period allowed the assessment of temporal trends in treatment use during a time of rapid evolution of treatment guidelines for advanced NSCLC. We stratified the cohort into different categories to reflect disease stages based on metastasis and surgery records.

Potential limitations of this study relate to the JMDC database, which only includes employed individuals and their families. Individuals who cannot work are not covered under JMDC insurance. This means that patients with the most severe disease, employees over 65 years of age, and family members older than 75 years were not captured. The enrolment of patients with advanced disease was likely to be limited as a result, which is a potential source of bias in our study. Nevertheless, the incidence rates of lung cancer in the JMDC population, and the age and sex distribution of patients with lung cancer, have been shown to accurately reflect data from the Japan National Cancer Registry when detection algorithms employ a combination of diagnosis, procedure, and medicine codes, as conducted in our study [22]. The JMDC has been used previously to evaluate survival, treatment safety outcomes, and treatment cost-effectiveness in lung cancer [23,24,25]. Procedures and drugs in the JMDC were defined based on claims records for reimbursement purposes, and clinical and staging data are not captured. The inaccuracy of these data could have led to the misclassification of patients into different groups in our study [26].

## 5. Conclusions

In conclusion, we observed marked changes in the treatment of advanced lung cancer between 2015 and 2023, driven by a rapid increase in the use of targeted therapies including checkpoint inhibitors. These changes appear to be aligned with current treatment guidelines in Japan. Fewer patients showed evidence of distant metastases over time, possibly indicating earlier detection. Additional research to understand if access to targeted therapies is also being extended to older and frail patients with lung cancer in Japan is needed.

## Figures and Tables

**Table 1:** Patient demographic and clinical characteristics during the baseline period stratified by severity grouping.

	S (N = 5479)		NS-NM (N = 2084)		NS-RM (N = 375)		NS-DM (N = 1435)	
Sex, *n* (%)								
Female	1966	(35.98)	633	(30.4)	116	(30.9)	423	(29.5)
Male	3513	(64.1)	1451	(69.6)	259	(69.1)	1012	(70.5)
Age (years)								
Median (Q1, Q3)	59.59	(53.09, 65.11)	60.94	(53.66, 66.06)	60.59	(53.96–66.31)	59.62	(53.08–65.05)
Baseline CCI score *n* (%)								
0	3692	(67.4)	1282	(61.5)	242	(64.5)	969	(67.5)
1	982	(17.9)	379	(18.2)	66	(17.6)	193	(13.4)
2	448	(8.2)	179	(8.6)	33	(8.8)	92	(6.4)
3	184	(3.4)	111	(5.3)	8	(2.1)	44	(3.1)
4+	173	(3.2)	133	(6.4)	26	(6.9)	137	(9.6)
Baseline * BMI *n* (%)								
<18.5	377	(6.9)	165	(7.9)	32	(8.5)	96	(6.7)
18.5–<24	2850	(52.0)	924	(44.3)	148	(39.5)	612	(42.7)
≤24	1712	(31.3)	566	(27.2)	101	(26.9)	409	(28.5)
Missing	540	(9.9)	429	(20.6)	94	(25.1)	318	(22.2)
Smoker, *n* (%)								
Yes	1355	(24.7)	717	(34.4)	119	(31.7)	477	(33.2)
No	3571	(65.2)	932	(44.7)	161	(42.9)	640	(44.6)
Missing	553	(10.1)	435	(20.9)	95	(25.3)	318	(22.2)
Alcohol consumption, *n* (%)								
Everyday	1660	(30.3)	582	(27.9)	102	(27.2)	417	(29.1)
Sometimes	1293	(23.6)	392	(18.8)	66	(17.6)	257	(17.9)
Rarely	1878	(34.3)	625	(30.0)	106	(28.3)	419	(29.2)
Missing	649	(11.9)	485	(23.3)	101	(26.9)	342	(22.8)

BMI—body mass index; CCI—Charlson comorbidity index; SD—standard deviation. S group: patients with at least one record of surgery; NS-NM group: patients with no record of surgery and no metastasis; NS-RM group: patients with no record of surgery but at least one record of regional metastasis; NS-DM group: patients with no record of surgery but at least one record of distant metastasis. * closet to baseline

**Table 2:** Time to treatment initiation and duration of initial treatment episode.

	S (N = 5479)	NS-NM (N = 2084)	NS-RM (N = 375)	NS-DM (N = 1435)
Days from diagnosis to treatment initiation				
Median (Q1, Q3)	38 (22, 58)	34 (19, 69.5)	33 (19, 56)	25 (14, 40)
Time to initial treatment episode, *n* (%)				
≤30 days	1938 (37.7)	954 (45.8)	228 (43.3)	945 (58.1)
>30 to ≤60 days	2039 (39.7)	544 (26.1)	166 (31.5)	461 (28.4)
>60 days	1160 (22.6)	586 (28.1)	133 (25.2)	219 (13.5)
Duration of initial treatment episode (days)				
Median (Q1, Q3)	1 (1, 155)	196 (77, 473)	238 (119, 472)	260 (109, 523)
Duration of initial treatment episode, *n* (%)				
0 to ≤3 months	3457 (67.3)	582 (27.9)	138 (26.2)	390 (24.0)
>3 to ≤6 months	702 (13.7)	406 (19.5)	123 (23.3)	294 (18.1)
>6 months	978 (19.0)	1096 (52.6)	266 (50.5)	941 (57.9)

Q1, Q3—first and third quartiles; SD—standard deviation. S group: patients with at least one record of surgery; NS-NM group: patients with no record of surgery and no metastasis; NS-RM group: patients with no record of surgery but at least one record of regional metastasis; NS-DM group: patients with no record of surgery but at least one record of distant metastasis.

**Table 3:** Single or multiple treatment strategies in initial treatment episodes, *n* (%).

	S (N = 5479)	NS-NM (N = 2084)	NS-RM (N = 375)	NS-DM (N = 1435)
Monotherapy	3251 (59.3)	467 (22.4)	98 (26.1)	350 (24.4)
Surgery alone	3251 (59.3)	-	-	-
Chemotherapy	-	224 (10.7)	44 (11.7)	96 (6.7)
Checkpoint inhibitors/targeted therapy	-	156 (7.5)	47 (12.5)	145 (10.1)
RT	-	87 (4.2)	7 (2.9)	109 (17.4)
Combined treatments	2228 (40.7)	1617 (77.6)	277 (73.9)	1085 (75.6)
Surgery + chemotherapy	1508 (27.5)	-	-	-
Surgery + checkpoint inhibitors/targeted therapy	48 (0.9)	-	-	-
Surgery + RT	25 (0.5)	-	-	-
Surgery + chemotherapy + checkpoint inhibitors/targeted	163 (3.0)	-	-	-
Surgery + chemotherapy + RT ± checkpoint inhibitors/targeted	277 (5.1)	-	-	-
Surgery + checkpoint inhibitors/targeted + RT	7 (0.1)			
Surgery + other systemic therapy	200 (3.7)			
Chemotherapy + checkpoint inhibitors/targeted	-	360 (17.3)	118 (31.5)	216 (15.1)
Chemotherapy + RT ± checkpoint inhibitors/targeted	-	782 (37.5)	123 (32.8)	676 (47.1)
Concurrent chemoradiotherapy	-	672 (85.9)	97 (78.9)	447 (66.1)
Sequential chemoradiotherapy	-	110 (14.1)	26 (21.1)	229 (33.9)
Checkpoint inhibitors/targeted + RT	-	36 (1.7)	6 (1.6)	132 (9.2)
Other systemic therapy	-	439 (21.1)	30 (8.0)	61 (4.6)

PD-(L)1—programmed cell death (ligand) 1; RT—radiotherapy. “Other systemic therapy” includes immunosuppressants, hormones and related agents, and immunostimulants. S group: patients with at least one record of surgery; NS-NM group: patients with no record of surgery and no metastasis; NS-RM group: patients with no record of surgery but at least one record of regional metastasis; NS-DM group: patients with no record of surgery but at least one record of distant metastasis.

## Data Availability

The data underlying this article were provided by the JMDC under license. Data will be shared on request to the corresponding author with the permission of the JMDC.

## Supplementary Materials

The following supporting information can be downloaded at https://www.mdpi.com/article/10.3390/curroncol32010032/s1, Table S1: Radiotherapy codes and terms in the JMDC. Table S2: Surgery codes and terms in the JMDC. Table S3: Codes and definitions for chemotherapy codes, PD-1/PD-L1, and targeted therapy. Table S4: ICD-10-CM codes used to identify metastases. Figure S1: Selection of the study analysis population.
