# Supplementary material for: Initial Treatment Modalities in Patients with Newly Diagnosed Primary Lung Cancer in Japan
Source: Curr Oncol. 2025 Jan 7;32(1):32. doi: 10.3390/curroncol32010032 (PMC11763771; doi:10.3390/curroncol32010032)
Supplement: Supplementary file 1 [file curroncol-32-00032-s001.zip › curroncol-3362316-supplementary.pdf]

# Initial Treatment Modalities in Patients with Newly Diagnosed Primary Lung Cancer in Japan

## SUPPLEMENT

**Table S1**. Radiotherapy codes and terms in the JMDC

| M code | Procedure name | Procedure code |
|---|---|---|
| M001 | external irradiation (superficial x-ray radiotherapy) (first time) | 180008810 |
| M001-2 | stereotactic radiotherapy with gamma knife | 180018910 |
| M001-3 | radiotherapy with linear accelerator (stereotactic radiotherapy) | 180019710 |
| M001 | external irradiation (high energy radiotherapy, 2 opposing field irradiation) (first time) | 180020810 |
| M001 | external irradiation (high energy radiotherapy, 2 non-opposing field irradiation) (first time) | 180020910 |
| M001 | external irradiation (high energy radiotherapy, 3 field irradiation) (first time) | 180021010 |
| M001 | external irradiation (high energy radiotherapy, 4 field or more irradiation) (first time) | 180021110 |
| M001 | external irradiation (high energy radiotherapy, moving field irradiation) (first time) | 180021210 |
| M001 | external irradiation (high energy radiotherapy, conformal irradiation) (first time) | 180021310 |
| M001 | external irradiation (high energy radiotherapy, single field irradiation) (second time) | 180021410 |
| M001 | external irradiation (high energy radiotherapy, 2 opposing field irradiation) (second time) | 180021510 |
| M001 | external irradiation (high energy radiotherapy, 2 non-opposing field irradiation) (second time) | 180021610 |
| M001 | external irradiation (high energy radiotherapy, 3 field irradiation) (second time) | 180021710 |
| M001 | external irradiation (high energy radiotherapy, 4 field or more irradiation) (second time) | 180021810 |
| M001 | external irradiation (high energy radiotherapy, moving field irradiation) (second time) | 180021910 |
| M001 | external irradiation (high energy radiotherapy, conformal irradiation) (second time) | 180022010 |
| M001 | subtraction on facility standard non-conformity (70/100) [radiotherapy] | 180025270 |
| M001-3 | radiotherapy with linear accelerator (stereotactic radiotherapy, for trunk) | 180026750 |
| M001 | external irradiation (intensity modulated radiotherapy, IMRT) | 180031910 |
| M001 | external irradiation (intensity modulated radiotherapy, IMRT) (second time) | 180032010 |
| M001-3 | radiotherapy with linear accelerator | 180035310 |
| M001-4 | ion beam radiotherapy (baryon beam) | 180046610 |
| M001-4 | ion beam radiotherapy (proton beam) | 180046710 |
| M001-4 | ion beam radiotherapy (boron neutron capture therapy) | 180064350 |
| M001 | [J] external irradiation [2-a-1], high energy radiotherapy, single field irradiation or 2 opposing field irradiation (first time) | 999900467 |
| M001 | [J] external irradiation [2-a-2], high energy radiotherapy, 2 non-opposing field irradiation or 3 field irradiation (first time) | 999900468 |

| | | |
|---|---|---|
| M001 | [J] external irradiation [2-a-3], high energy radiotherapy, 4 field or more irradiation, moving field irradiation/conformal irradiation (first time) | 999900469 |
| M001 | [J] external irradiation [3-b-1], high energy radiotherapy, single field/2 opposing field irradiation (second time) | 999900470 |
| M001 | [J] external irradiation [3-b-2], high energy radiotherapy, 2 non-opposing field/3 field irradiation (second time) | 999900471 |
| M001 | [J] external irradiation [2-b-3], high energy radiotherapy, 4 field or more irradiation, moving field irradiation/conformal irradiation (second time) | 999900472 |

**Table S2**. Surgery codes and terms in the JMDC.

| K code | Procedure name | Procedure code |
|---|---|---|
| K511 | lung resection (partial cuneiform resection) | 150129710 |
| K511 | lung resection, segmental resection (smaller than a single lobe) | 150129810 |
| K511 | lung resection (lobectomy) | 150129910 |
| K511 | lung resection, composite resection (larger than a single lobe) | 150130010 |
| K511 | lung resection (whole lung on one side) | 150130110 |
| K511 | lung resection, combined with thoracoplasty | 150130650 |
| K514 | malignant lung tumor surgery (resection of a whole lobe or smaller than a single lobe) | 150130810 |
| K514 | malignant lung tumor surgery (resection of whole lung on one side or larger than a single lobe) | 150130910 |
| K513 | thoracoscopic lung resection (pulmonary cyst surgery, partial cuneiform resection) | 150266610 |
| K514 | malignant lung tumor surgery (lung resection with bronchoplasty) | 150317210 |
| K513 | thoracoscopic lung resection (other) | 150357710 |
| K514 | malignant lung tumor surgery (partial resection) | 150357810 |
| K514 | malignant lung tumor surgery (segmental resection) | 150357910 |
| K514 | malignant lung tumor surgery (resection of a whole lobe or larger than a single lobe) | 150358010 |
| K514 | malignant lung tumor surgery (lung resection with combined resection of adjacent organ) | 150358210 |
| K514 | malignant lung tumor surgery (lung resection with bronchoplasty) | 150358310 |
| K514 | malignant lung tumor surgery (lung resection with tracheal bifurcation reconstruction) | 150358510 |
| K514-2 | thoracoscopic malignant lung tumor surgery (partial resection) | 150358610 |
| K514-2 | thoracoscopic malignant lung tumor surgery (segmental resection) | 150358710 |
| K514-2 | thoracoscopic malignant lung tumor surgery (resection of a whole lobe or larger than a single lobe) | 150358810 |
| K514 | malignant lung tumor surgery (including parietal/visceral pleura, with combined resection of diaphragm and pericardium) | 150386610 |
| K514-2 | thoracoscopic malignant lung tumor surgery (resection of a whole lobe or larger than a single lobe) (using supporting device for endoscopic surgery) | 150406110 |
| K513 | thoracoscopic lung resection (partial resection) | 150414110 |

| | | |
|---|---|---|
| K513 | thoracoscopic lung resection (segmental resection) | 150414210 |
| K513 | thoracoscopic lung resection (resection of a whole lobe or larger than a single lobe) | 150414310 |
| K514-2 | thoracoscopic malignant lung tumor surgery (segmental resection) (using supporting device for endoscopic surgery) | 150414410 |

**Table S3**. Codes and definitions for chemotherapy codes, PD-1/PD-L1, and targeted therapy.

| **Groups** | **Definition** | **ATC Codes** |
|---|---|---|
| Chemotherapy | **Platinum:** e.g. cisplatin, carboplatin, oxaliplatin, nedaplatin, and lobaplatin<br>**Non-platinum:** e.g. gemcitabine, paclitaxel, pemetrexed, docetaxel, etoposide, vinorelbine tartrate, doxorubicin, methotrexate sodium, Irinotecan, Vinblastine, Vincristine, Vinorelbine<br>UFT (uracil, tegafur)<br>S-1 (tegafur, gimestat, otastat)<br>Bendamustine, cyclophosphamide, Ifosfamide, Dacarbazine | Platinum: ATC L01XA Platinum compounds<br>Non-platinum: ATC L01B antimetabolites; ATC L01C plant alkaloids and other natural products; ATC L01D cytotoxic antibiotics and related substances.<br>ATC L01A ALKYLATING AGENTS<br>ATC L01X OTHER ANTINEOPLASTIC AGENTS |
| PD1/PD-L1 | Nivolumab, pembrolizumab, cemiplimab, atezolizumab, durvalumab, avelumab, tislelizumab<br>spartalizumab, camrelizumab, sintilimab, toripalimab, dostarlimab | ATC L01FF PD-1/PDL-1 (Programmed cell death protein 1/death ligand 1) inhibitors |
| Target therapy | **Small-molecule drugs**, e.g., erlotinib, afatinib, gefitinib, osimertinib, dacomitinib, crizotinib, mobocertinib, ceritinib, alectinib, brigatinib or lorlatinib<br>cabozantinib, capmatinib, dabrafenib entrectinib, larotrectinib, pralsetinib, selpercatinib, tepotinib, and trametinib, entrectinib Capmatinib, Tepotinib, Brigatinib<br>**Monoclonal antibodies:** Ramucirumab L01FG02 Trastuzumab emtansine L01FD03 Bevacizumab L01FG01 Ipilimumab L01FX04, Mogamulizumab L01FX09 | ATC L01E protein kinase inhibitors<br>ATC L01F MONOCLONAL ANTIBODIES AND ANTIBODY DRUG CONJUGATES (except L01FF) |

**Table S4**. ICD-10-CM codes used to identify metastases

| **Distant metastases** | **Regional metastases** |
|---|---|
| Lymph nodes C77.2-77.9<br>Abdomen C78.4–78.6, C78.8<br>Liver C78.7<br>Bone C79.5<br>Brain C79.3<br>Other sites C79.0–79.2, C79.4, C79.6-C79.9 | C77.0 Neck lymph nodes<br>C77.1 Intrathoracic lymph nodes–bronchopulmonary, intercostal, mediastinal, tracheobronchial<br>C78.0 Lung Bronchus<br>C78.1 Mediastinum<br>C78.2 Pleura<br>C78.3 Other and unspecified respiratory organs |

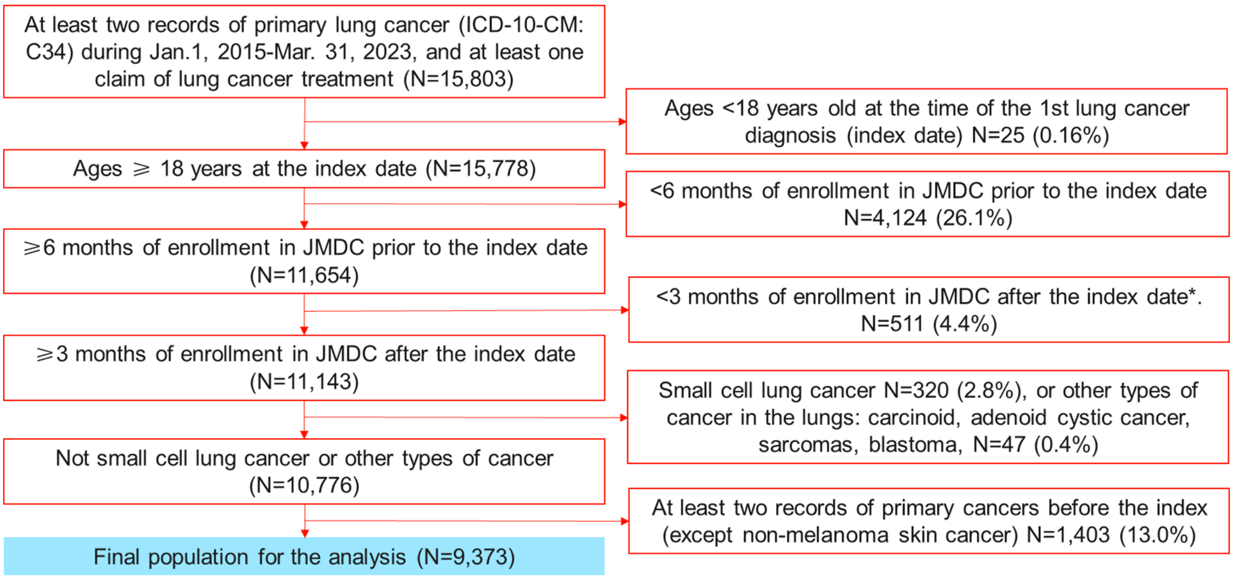


**Figure S1.** Selection of the study analysis population
